# Installation of the developing nephron in the fetal human kidney during advanced pregnancy

**DOI:** 10.1186/s40348-023-00172-4

**Published:** 2023-11-28

**Authors:** Will W. Minuth

**Affiliations:** Institute of Anatomy, University of Regensburg, D-93053 Regensburg, Germany

**Keywords:** Fetal human kidney, Nephrogenic zone, Nephron development, Interstitium, Installation, Impairment of nephrogenesis

## Abstract

**Background:**

The kidneys of preterm and low birth weight babies reflect vulnerability, since several noxae can evoke the termination of nephron formation. This again leads to oligonephropathy with severe consequences for health in the later life. While the clinical parameters have been intensely investigated, only little is known about the initial traces left by the noxae. For the fetal human kidney, solely the lack of basophilic S-shaped bodies and the reduction in width of the nephrogenic zone were registered. It is not known in how far also the involved progenitor cells, the earlier nephron stages, the collecting duct (CD) ampullae, and the local interstitium are collaterally harmed.

**Aim:**

The interstitium at the forming nephron is heterogeneously structured. Thereby, it fulfills quite different mastering and integrative tasks. Since data dealing with the installation of a nephron is not available, the microanatomical features were recorded.

**Results:**

The microscopic specimens show that the installation of the transient stages of nephron anlage is not synchronized. Instead, it is controlled within a nephrogenic compartment of the nephrogenic zone. It starts near the renal capsule by positioning the nephrogenic niche so that the nephrogenic progenitor cells face the epithelial progenitor cell at the tip of a CD ampulla. Then, the induced nephrogenic progenitor cells assimilate in the pretubular aggregate. While its medial part remains opposite the head of the CD ampulla, at its proximal end, the primitive renal vesicle is formed. Only a part of it separates to stick to the section border between the head and conus of the CD ampulla. This marks the link with the future connecting tubule at the distal pole of the extending renal vesicle. Meanwhile, the proximal pole is mounted next to the connecting tubule of an earlier developed nephron. The resulting two-point mounting serves a common elongation of the conus at the CD ampulla and the medial aspect of the comma-shaped body. In the S-shaped body, it supports to defoliate the arising glomerulus and to link it with the perforating radiate artery at its deep lateral aspect.

**Conclusions:**

The investigation depicts that the installation is an interactive process between the stages of nephron anlage and its structural neighbors. A special meaning has the interjacent interstitium. It is vital for the positioning, shaping, and physiological integration. Due to its special location, this is mainly exposed to noxae.

## Background

Although the kidneys are of paramount physiological importance to the organism, many questions dealing with their structural development and the etiology of related diseases are still unanswered. This is particularly reflected not only in basic research but also in the area of pediatric nephrology. For example, one of the peculiarities of the fetal human kidney is that the process of nephrogenesis is not evenly running but that it accelerates. More than half of the total number of nephrons arises in the last 3 months of pregnancy [1]. The clinical experiences with preterm and low birth weight babies indicate that the kidneys are vulnerable in this phase of increasing developmental activity, since a series of noxae such as malnutrition, diabetes, vitamin deficiency of the mother, insufficiency of the placenta, hyperoxia and also drugs can evoke the termination of nephrogenesis [2]. This again leads to oligonephropathy/oligonephronia with severe consequences for health in the later life [3–6].

On a closer look, there is a striking imbalance between the numerous clinical experiences with babies showing harmed kidneys, the only few pathological findings, the lack of knowledge dealing with the cellular and molecular targets attacked by the harming, and the up to date missing therapeutic options. In this context, the screening of literature points out that for a long time the related research, which is exclusively focusing on the conditions in the fetal human kidney during late pregnancy, has been neglected. To give a typical example, only few pathological findings were reported, which are dealing with the initial damage left by the noxae impairing nephrogenesis. Certainly, it is known that the noxae aim at the outer cortex in the kidneys of preterm and low birth weight babies. However, for the externally situated nephrogenic zone, only the reduction of its vertical width [7] and the loss of only here occurring basophilic S-shaped bodies [8] were recorded. This points out that the first links in the chain of nephron formation are damaged. For the subjacent maturation zone, a reduced number as the occurrence of atypical glomeruli, which exhibit an extended Bowman’s space and a shrunken glomerular tuft, were registered [9]. This indicates that beside the first also the later links of nephron formation can be harmed. However, even important data dealing with the structural neighbors were not communicated. Consequently, it is not known, whether the before mentioned noxae evoke the same sort of damage, or whether there might be different patterns of harming in the nephrogenic zone.

It is obvious that the lack of knowledge can only be resolved by performing basic research work. However, quite surprising is that the search for initial cellular and molecular traces left by the noxae has been performed solely by different animal models, while unfortunately it was not verified with samples of the fetal human kidney [10–13]. Due to the registered morphological, cell, and molecular biological species differences, it is questionable in this specific issue, whether or to which degree the results generated in animal models are readily transferable to the fetal human kidney [14, 15]. Modern tissue culture experiments with organoids derived from the nephrogenic zone of the fetal human kidney may be a more appropriate solution.

A further obstacle for the search of initial traces left by harming is that a solid microanatomical base for the outer cortex of the fetal human kidney during advanced pregnancy was not available. Only during the last few years, first information about the specific morphological features of the nephrogenic zone [16, 17], the shaping of the nephron [18], the mutual patterning with its structural neighbors [19], and the different compartments of the local interstitium [20] were introduced. Related systematic ultrastructural data generated by modern transmission and scanning electron microscopical methods is not yet available. For these reasons, it is not surprising that urgently needed information dealing for example with the developing microvasculature is barely known and that basic physiological data dealing with the composition of local nutrition, the content of respiratory gases, the buffer system, the regulation of pH, and the circulating metabolites in the nephrogenic zone of the fetal human kidney during advanced pregnancy is completely lacking.

A real challenge for the search of initial traces left by noxae is that quite different plains of molecular regulation in the fetal human kidney exist, by which the formation of nephrons is controlled. For example, the average number of nephrons in an adult human kidney is approximately 1,000,000, but there is a variation. It can range from as low as 200,000 to as high as 2.7 million [21, 22]. The resulting endowment reflects the finally available number of nephrons in the adult organ, which again depends on a complex interplay between the personal renal genetic blueprint, the in utero situation, and the different environmental exposures [23, 24].

When the focus of analytical interest is directed to the formation of one of the nephrons in the fetal human kidney during advanced pregnancy, each of them must pass not only its own morphogenesis, at the same time it must be integrated into the radially expanding nephrogenic zone. While the molecular control of the involved nephrogenic progenitor cells, the actions of the different morphogenic proteins, the nephron patterning, and the resulting endowment was subject of intense research, data is not available dealing with the installation of a nephron and its integration into the structural neighbors [25, 26].

By the first view, the installation of the forming nephron appears to be a simple radial apposition of the successively developing stages of nephron anlage onto maturing nephron structures. However, a closer look reveals that it is a complex and to date not in detail investigated own process. So far, one can see that it starts next to the inner side of the renal capsule, lines vertically und bidirectionally through the nephrogenic zone, proceeds within predictable coordinates, and takes place in close relation along the different sectors of the related collecting duct (CD) ampulla [19]. Thereby, a series of basic morphological settings are realized, which includes for example the lasting position of the forming nephron, the available space for the prospective spatial expansion, the physiological connection between the future connecting tubule and the CD ampulla, and finally the microvascular linking between the tuft of the arising glomerulus and the neighboring perforating radiate artery. One must realize that principally each of these crucial sites may be a target of the earlier mentioned noxae.

While the formation of a nephron takes place, not only the neighboring CD ampulla or the perforating radiate artery but also the interjacent interstitium are interactively involved. However, little noticed is that the different compartments of the local interstitium fulfill not only piloting but also important integrative tasks [27–29]. An indispensable part of them is the here running process of installation. To generate information about this uncharted issue and to support the search for initial traces left in harmed kidneys, the present microanatomical analysis was performed.

## Material and methods

In order to obtain a morphological survey about the installation of the forming nephron, one has to focus on the transient stages of nephron anlage such as the nephrogenic niche, pretubular aggregate, mesenchymal to epithelial transition, renal vesicles, comma-, and S-shaped bodies. In the fetal human kidney during advanced pregnancy, these are restricted to the nephrogenic zone [17]. Since it lines along the entire inner side of the renal capsule, a damage of this particular region during the histological preparation must be prevented. As a consequence, a fetal human kidney is held only on its hilum so that the touching of the renal capsule with fine forceps is avoided. Further on, for obtaining comparable perspectives in the histological sections, a fixed kidney is cut from the renal capsule towards the papilla of a lobe. Following this advice, the section plane lines along the axis of the vertically running collecting duct tubules and at the same time perpendicular to the renal capsule.

For the here shown illustrations, specimens of the fetal human kidneys of gestational age between week 16 to 18 and later were selected from the stock of preparations used for the Course of Microscopic Anatomy for Medical Students at the University of Regensburg in Germany. These stages are insofar of special interest, because up until birth, the majority of the nephrons is formed in the expanding organ.

According to routine methods, the tissue blocks were fixed in paraformaldehyde solution and embedded in paraffin wax. Then, sections of 5 μm thickness were cut and stained with hematoxylin-eosin solution for analysis by the optical microscope. The specimen shown in Fig. 10 was kindly provided by Professor Dr. Klaus Tiedemann († 2023), Institute of Anatomy, University of Heidelberg, Germany. It was stained by Azan to show vessels, which are perfused by erythrocytes and vessels lacking erythrocytes. Screening of the sections was performed by a Leica DM750 microscope (Leica Microsystems, Wetzlar, Germany). The single stages of nephron anlage were analyzed with a HI Plan ×63/0.75 objective lens. Images were taken with a Basler Microscopy Pulse 5.0 camera (Basler AG, Ahrensburg, Germany).

More than 3000 images were available, which had been analyzed for earlier performed investigations dealing with the shaping [18], the mutual patterning of the nephron with its structural neighbors [19], and the interjacent interstitium [20]. From this stock, those representative images were selected, which illustrate the basic stations of the initial nephron development.

For depicting and clear comprising the temporal and spatial alterations during the installation process, beside the photographic illustrations, a series of graphical sketches almost true to scale were produced. To meet the defined criteria, the borders between the individual stages of nephron anlage and their covering tissues were marked 1:1 by a pencil by the hand. In a further step, the generated contours were scanned, edited, and processed by the design program CorelDRAW 2021 (Corel Corporation, Munich, Germany). For inserting the necessary labels and for obtaining information about the metric parameters in the microscopic images or produced sketches, the same program was used.

To visualize the morphological changes during the process of installation, original photographic images and graphical sketches almost true to scale are presented. It is indicated that the proximal pole (mounted point) of the currently developing nephron stage rests next to the connecting tubule of a previously developed nephron. Since the space is limited at this site, the distal pole (mobile point) of the currently developing stage of nephron anlage vertically shifts in parallel with the elongating conus of the CD ampulla during the further development. Thereby, it radially postpones the overlying pool of progenitor cells and the renal capsule.

## Results

### Technical versus dynamic installation

**Fig. 1:**
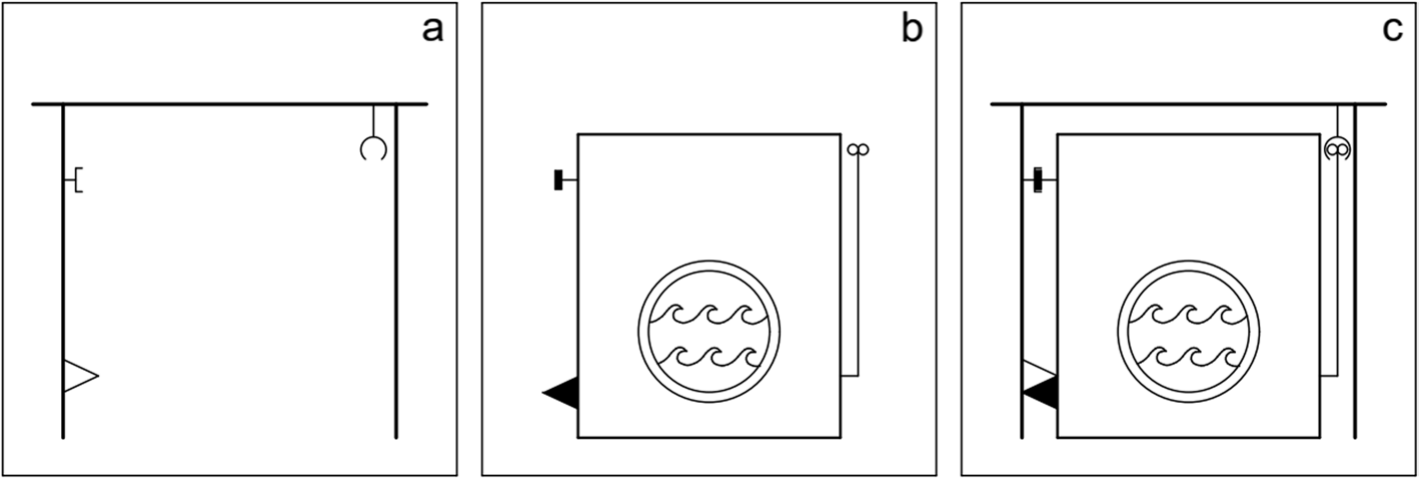
**a**–**c** Schematic illustration depicts the technical installation of a washing machine. **a** The housing under the worktop has a constant size. **b** The machine to be installed has distinct measurements, a definitive form, and a certain number of connections. **c** The mounting can be completed, since the machine fits into the housing and the connections are at the right place

**Fig. 2:**
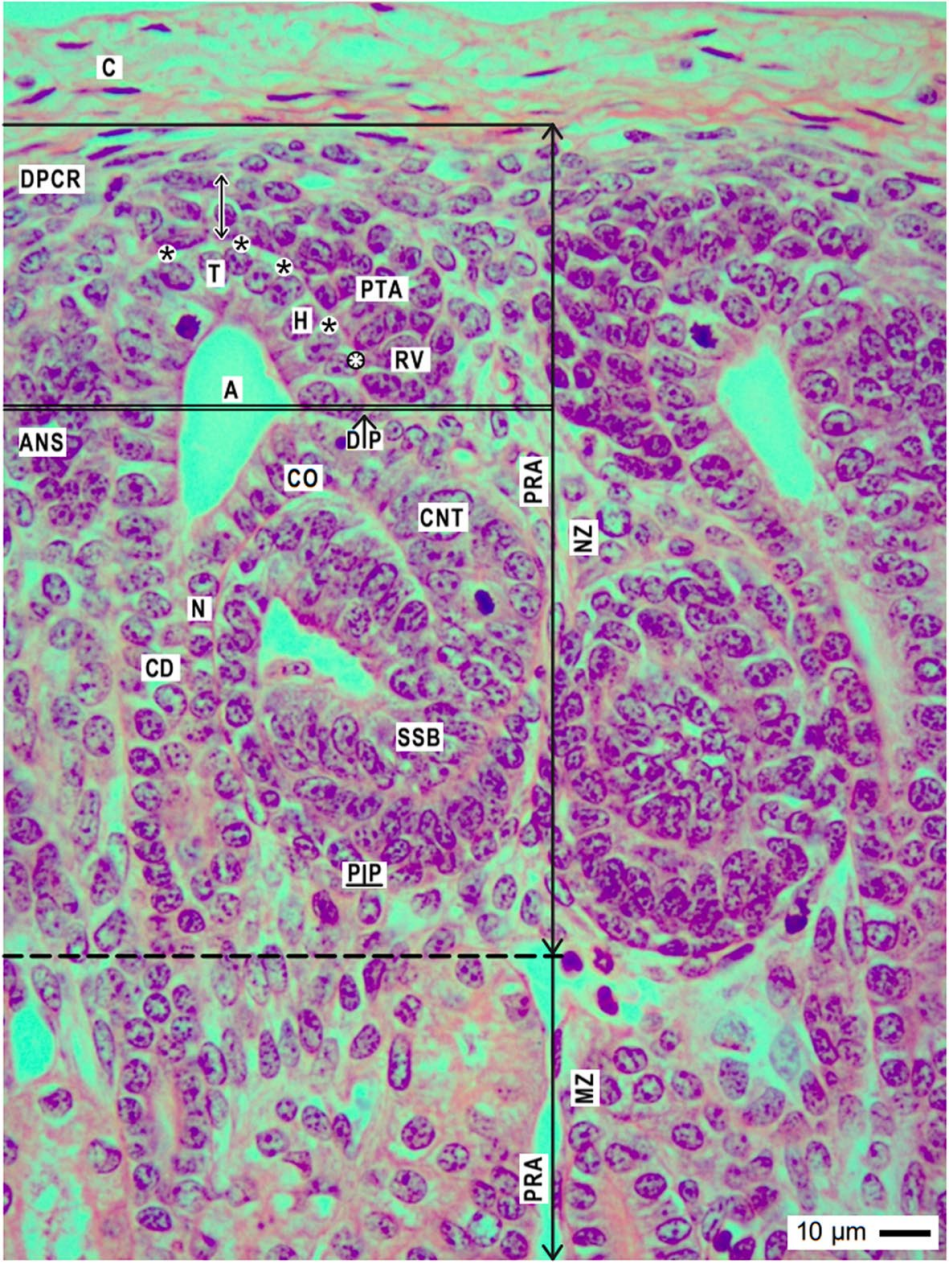
View onto the outer cortex of the fetal human kidney during advanced pregnancy by the optical microscope. Covered by the renal capsule (C), one can recognize the nephrogenic zone (NZ), and underneath the beginning maturation zone (MZ). Both are separated by a transverse dashed line. The vertical and transverse black lines indicate that the nephrogenic zone can be divided into side by side aligned nephrogenic compartments. In each, a collecting duct (CD) ampulla (A) is visible, which represents the dilated ureteric bud-derived end of the CD tubule. The CD ampulla consists of a tip (T), head (H), conus (CO), and neck (N). In the space between the inner side of the renal capsule and the tip of a CD ampulla (vertical double arrow), an outer layer of interstitial/stromal progenitor cells and 2 to 3 subjacent layers of nephrogenic progenitor cells are located. The innermost layer faces a clear interface (black asterisks). The pretubular aggregate (PTA) is positioned opposite the head of the CD ampulla. When a transverse double line is drawn along the section border between the head and conus of the CD ampulla to the meeting between the proximal end of the pretubular aggregate and the underlying connecting tubule (CNT) of an earlier developed nephron, the nephrogenic compartment can be subdivided. The upper part represents the district of progenitor cell recruitment (DPCR). Dependent on the developmental progress, in the subjacent area of nephron shaping (ANS), either a renal vesicle, a comma-, or a S-shaped body (SSB) is visible. Most relevant for the installation is the adhesion (white asterisks) of the proximal end at the pretubular aggregate on the CD ampulla, and the mounting of the future proximal pole at the henceforth shaping renal vesicle (RV) next to the connecting tubule of an earlier developed S-shaped body. The distance between its proximal (PP) and distal (DP) poles reflects the maximal vertical width of the area of nephron shaping. PRA vertically lining perforating radiate artery

**Fig. 3:**
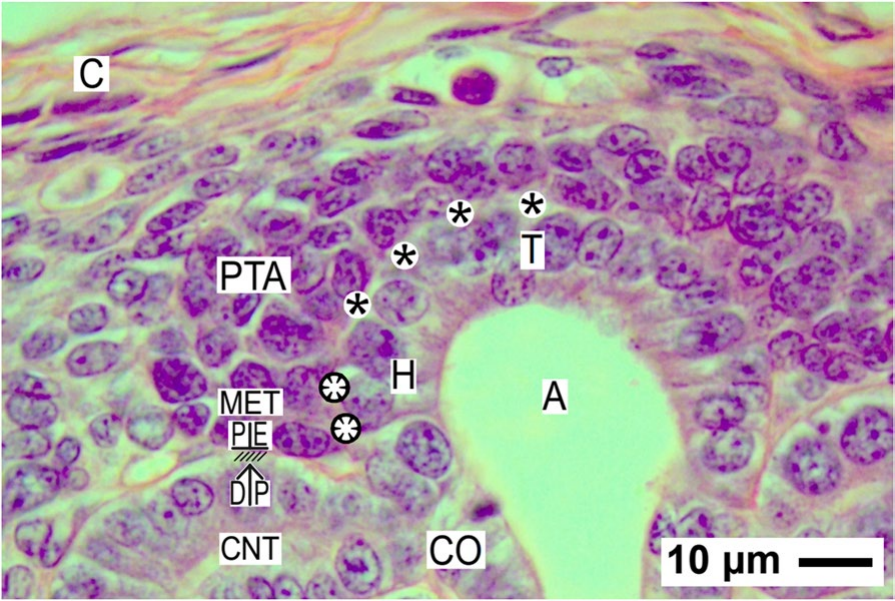
View onto the installation of the nephrogenic niche, the pretubular aggegate (PTA) and the mesenchymal to epithelia transition (MET) in the fetal human kidney during advanced pregnancy by the optical microscope. At the niche site (black asterisks), the innermost layer of the nephrogenic progenitor cells faces the epithelial progenitor cells, which are contained in the tip (T) of a CD ampulla (A). Between both cell types, a clear interface is visible. This extends up to the medial part of the pretubular aggregate (PTA). The lateral part of the pretubular aggregate is exposed to the subcapsular interstitium. The medial side of the proximal end (PE) at the PTA shows an adhesion (white asterisks) to the CD ampulla at the section border between its head (H) and conus (CO). The mid of the proximal end at the PTA indicates the proximal pole of the prospective nephron and its mounting (hatched) next to the connecting tubule (CNT) including the distal pole (DP) of a previously developed S-shaped body. Solely here, the mesenchymal to epithelial transition becomes active. The lateral side of the proximal end at the pretubular aggregate becomes positioned near a vertically lining perforating radiate artery. C renal capsule

**Fig. 4:**
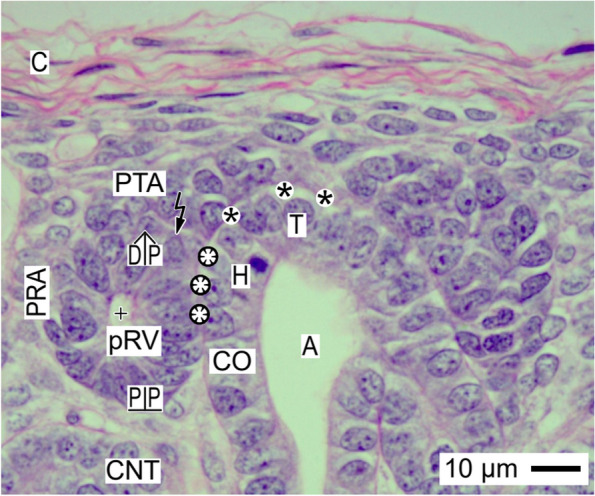
View onto the installation of the primitive renal vesicle (pRV) in the fetal human kidney during advanced pregnancy by the optical microscope. The installation supports that the primitive renal vesicle exclusively forms at the proximal end of the pretubular aggregate (PTA) by arranging the polarized epithelial cells around a small lumen (+). Meanwhile, at the distal pole (DP) of the primitive renal vesicle, a partial separation (flash) takes place so it temporary remains connected with the overlying pretubular aggregate. At its medial aspect, the primitive renal vesicle shows an adhesion (white asterisks), which begins at the head (H) to continue up to upper conus (CO) of the CD ampulla (A). The mounted proximal pole (PP) of the primitive renal vesicle is yet part of a pendentive, which is formed by the perivascular interstitium of the perforating radiate artery (PRA) and the connecting tubule (CNT) at a previously formed nephron. C renal capsule, black asterisks clear interface

**Fig. 5:**
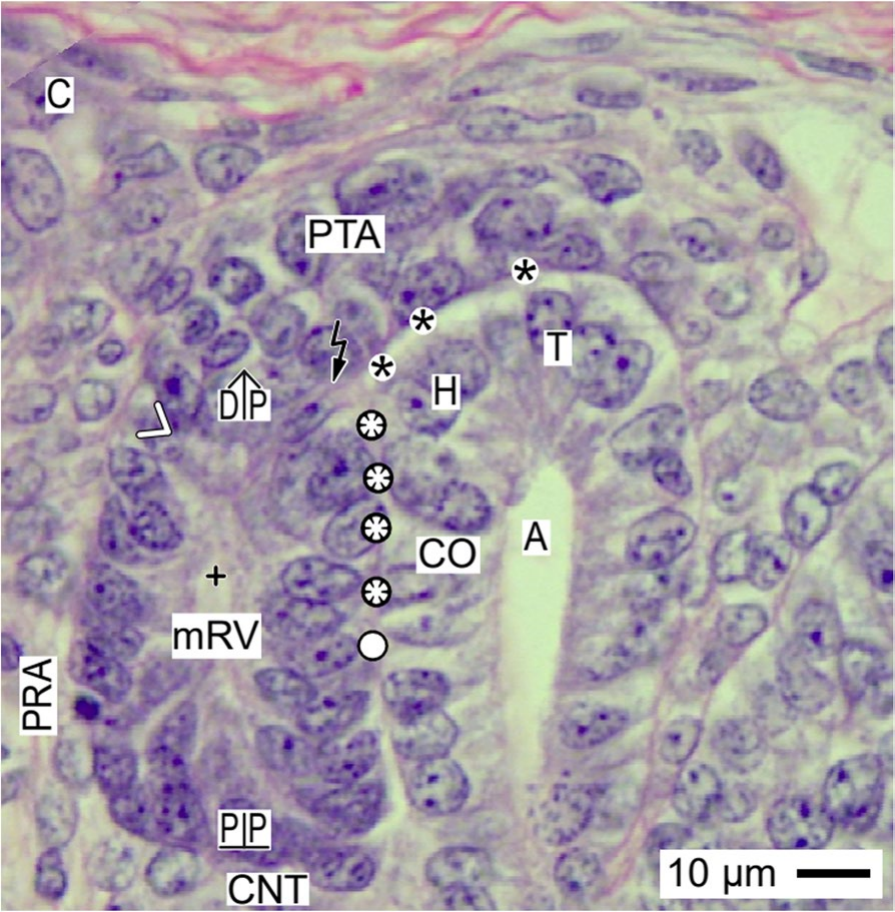
View onto the installation of the mature renal vesicle (mRV) in the fetal human kidney during advanced pregnancy by the optical microscope. While the mature renal vesicle arises, the local installation supports at its distal pole (DP) a partial separation (flash) from the overlying pretubular aggregate (PTA). This is visible at the end of the clear interface (black asterisks) and the adhesion (white asterisks). It is near the section border between the head (H) and conus (CO) of the CD ampulla (A). Underneath, the adhesion is replaced by a close interstitial cleft (white circle). During the further development, the medial aspect of the mature renal vesicle elongates in parallel with the upper conus of the CD ampulla. The lateral part of the distal pole at the mature renal vesicle remains connected with the proximal end of the PTA via a two-layered progenitor cell strand (white arrow head). C renal capsule, + lumen, PRA perforating radiate artery

**Fig. 6:**
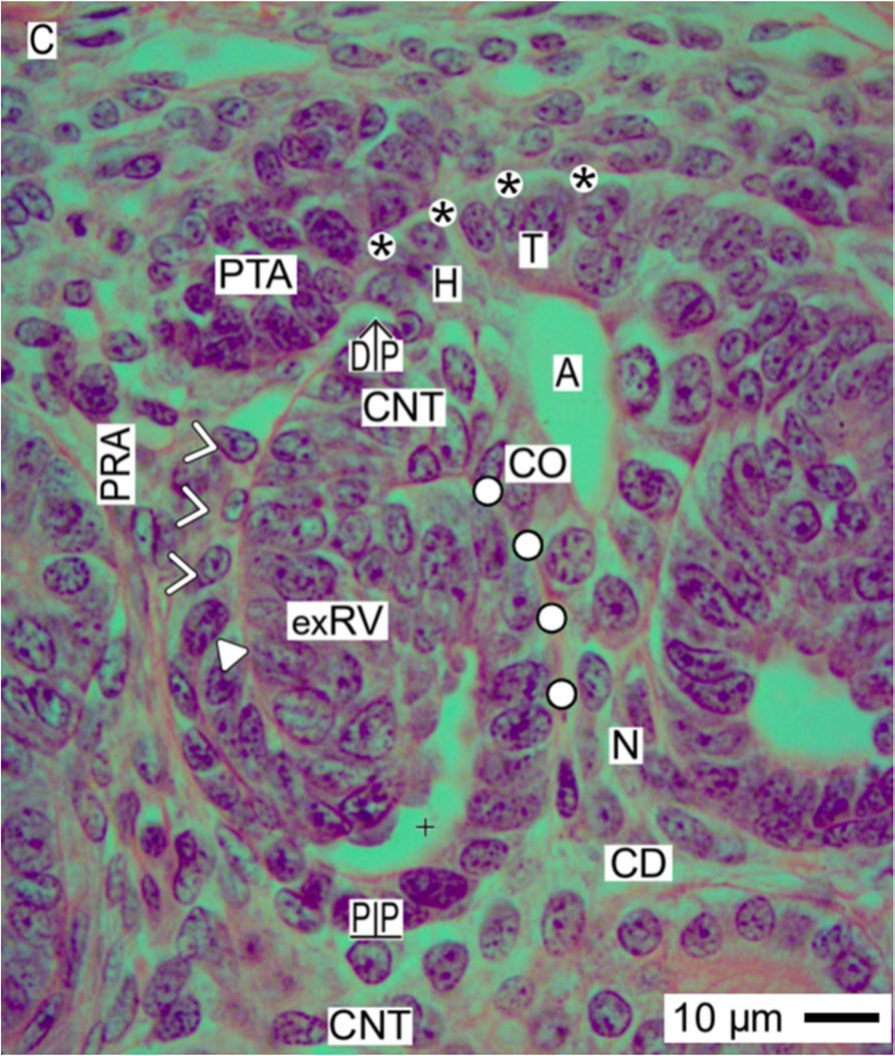
View onto the installation of the extending renal vesicle (exRV) in the fetal human kidney during advanced pregnancy by the optical microscope. Its distal pole (DP) with the establishing connecting tubule (CNT) is positioned next to the proximal end of the pretubular aggregate (PTA). Thereby, a pendentive is here formed by the subcapsular interstitium and the perivascular interstitium of a perforating radiate artery (PRA). At the same time, the connecting tubule invades the epithelium of the CD ampulla (A) between its head (H) and conus (CO). While the tubule anlage of the extending renal vesicle vertically elongates, the lateral aspect of the extending renal vesicle is still connected via a progenitor cell strand (white arrow heads) with the overlying pretubular aggregate. At the lateral fold, the formation of a cleft (white triangle) prepares the connection between the arising glomerular tuft and the vertically lining perforating radiate artery (PRA). At the medial aspect of the extending renal vesicle, the adhesion to the CD ampulla alters to a narrow interstitial cleft (white circles). C renal capsule, + lumen, black asterisks clear interface between the nephrogenic mesenchymal and epithelial progenitor cells in the niche, white asterisks adhesion, N neck of CD ampulla, CD collecting duct tubule, DP distal pole, PP proximal pole, C renal capsule

**Fig. 7:**
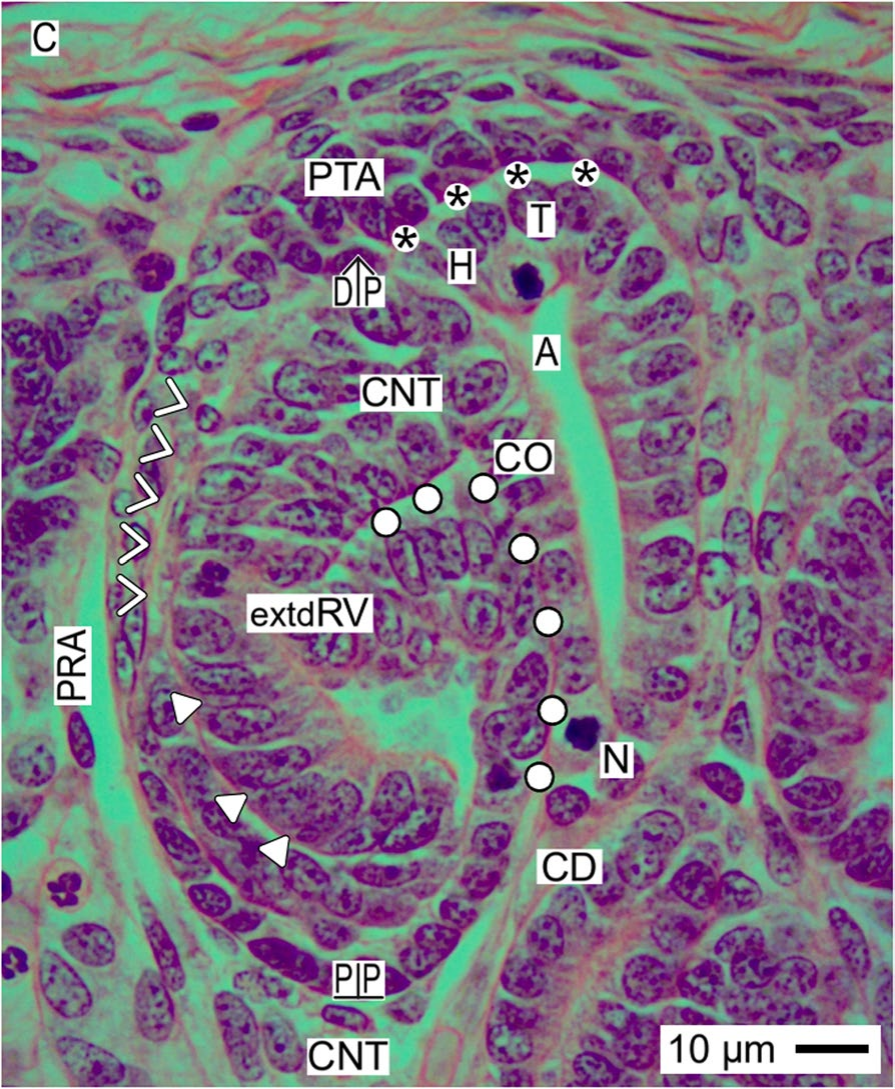
View onto the installation of the extended renal vesicle (extdRV) in the fetal human kidney during advanced pregnancy by the optical microscope. During this developmental phase, the vertical elongation of the CD ampulla (A) coincides with the internal folding. This causes that interstitial cleft lines from the connecting tubule (CNT) along the progenitor cell strand (white arrow heads) to the pretubular aggregate (PTA). While it elongates, it forms a deep oblique pocket. This step of installation enables the defoliation of the glomerulus (white triangles) at the lateral aspect of its proximal pole (PP). In contrast, the medial aspect of the extended renal vesicle shows a close course along the conus (CO) of the CD ampulla, which is later replaced by a narrow interstitial cleft (white circles). It expands in vertical direction. C renal capsule, PRA perforating radiate artery, black asterisk clear interface between the mesenchymal and epithelial progenitor cells in the nephrogenic niche, white asterisks adhesion, N neck of CD ampulla, CD collecting duct tubule, DP distal pole, C renal capsule

**Fig. 8:**
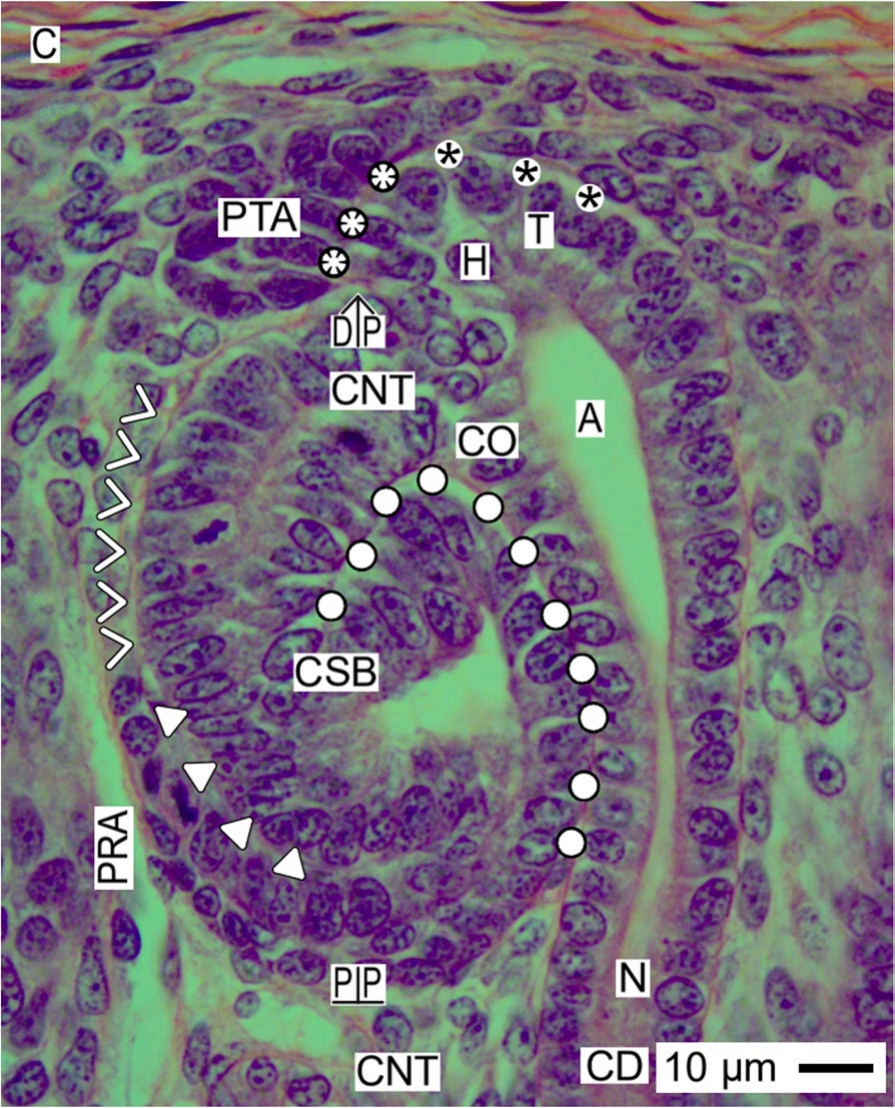
View onto the installation of the comma-shaped body (CSB) in the fetal human kidney during advanced pregnancy by the optical microscope. While the comma-shaped body is forming, the progenitor cell strand (white arrow heads), which lines to the proximal end of the pretubular aggregate (PTA), is dissolved by an unknown process during the proceeding installation. This causes that the neighboring peritubular interstitial pocket fuses with the perivascular interstitium at the neighboring perforating radiate artery (PRA). As a result, between the tubule anlage and the extending lateral fold, a cleft (white triangles) arises. It enables that the glomerulus defoliates at the proximal pole (PP) of the comma-shaped body. At the medial aspect, the narrow interstitial cleft (white circles) expands along the conus (CO) of the CD ampulla (A) towards the interior of the comma-shaped body. C renal capsule, black asterisks clear interface between the mesenchymal and epithelial progenitor cells in the nephrogenic niche, white asterisks adhesion, N neck of CD ampulla, CD collecting duct tubule DP distal pole, C renal capsule

**Fig. 9:**
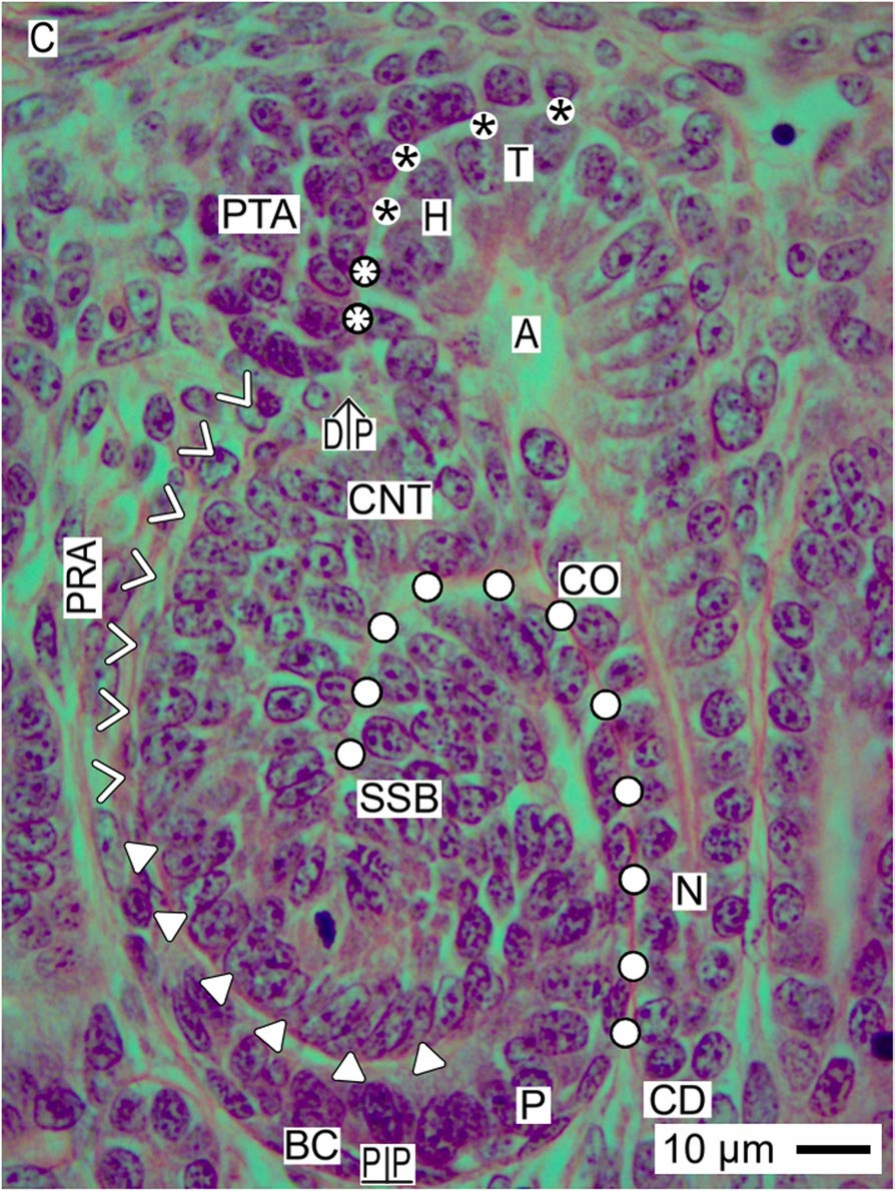
View onto the installation of the S-shaped body (SSB) in the fetal human kidney during advanced pregnancy by the optical microscope. Its connecting tubule (CNT) with the related distal pole (DP) is positioned near the proximal end of the regenerating pretubular aggregate (PTA). Depending on the environmental physiological conditions, at this meeting, a further nephron in form of a primitive renal vesicle can principally appear. At the lateral aspect, the elongating distal tubule is exposed to a neighboring perforating radiate artery (PRA). The progenitor cell strand (white arrow heads), which earlier lined to the pretubular aggregate, is yet dissolved by the installation. At the deep lateral aspect, the glomerulus defoliates along a narrow interstitial cleft (white triangles). This is bordered at the top by the expanding proximal tubule. Most decisively, it is invaded by interstitial cells so that as well as the afferent and efferent arterioles as the intra- and extraglomerular mesangium at the arising glomerulus can establish. Within the glomerulus, the visceral layer including the podocytes (P) and the parietal layer representing the Bowman’s capsule (BC) are visible. The later represents the proximal pole (PP), which is mounted next to the connecting tubule of a previously developed nephron. At the medial aspect of the S-shaped body, the vertical interstitial cleft (white circles) lines yet along the differentiating collecting duct (CD) tubule, the neck (N) and the conus (CO) of the CD ampulla (A) towards the center of the S-shaped body. The forming proximal, intermediate, distal, and connecting tubule portions become covered by an own faint peritubular interstitium C renal capsule, black asterisks interface between the nephrogenic mesenchymal and epithelial progenitor cells in the niche, white asterisks adhesion

To facilitate the entry into the unexpectedly complex issue of installation, a simple example and an illustrative tool are given. Nearly everyone can imagine the installation of a washing machine or a dish cleaner in the housing of a kitchen (Fig. 1). Typical for this technical installation is that the technical connections are present, that the housing underneath the worktop has the right size (Fig. 1a), that the machine has constant measurements, and that it exhibits a definitive form (Fig. 1b). It is obvious that only a professional installation enables the practical use of the appliance (Fig. 1c). In contrast, when the focus is directed to a developing nephron in the fetal human kidney during advanced pregnancy, the situation is completely different. As it will be shown in the Figs. 2, 3, 4, 5, 6, 7, 8 and 9, one part of the forming nephron to be installed stays mounted, while the other part radially shifts. Thereby, increasing sizes and altering shapes arise, which again are faced by progressively changing structural neighbors.

### Installation provides space

In the fetal human kidney during advanced pregnancy, the formation of new nephrons is restricted to the outer cortex. Covered by the renal capsule, it starts in the nephrogenic zone. This is subdivided into single nephrogenic compartments, which are aligned side by side, and in one row (Table 1, Fig. 2). In each of them, beside the formation of a single nephron, also its installation including the interactions with its structural neighbors such as the ureteric bud-derived CD ampulla and the perforating radiate artery can be recognized. Since the formation of the single nephrons is not synchronized, in each of the nephrogenic compartments, a different morphological composition is recognized. Always available structures are the CD ampulla, the perforating radiate artery, the pool of progenitor cells, the nephrogenic niche, and the related pretubular aggregate. In contrast, dependent on the individual developmental progress, either the mesenchymal to epithelia transition or one of the different renal vesicle stages, a comma-shaped body, or a S-shaped body is visible. The transition from the late S-shaped body to the maturing nephron is allocated to the subjacent maturation zone.

**Table 1:**
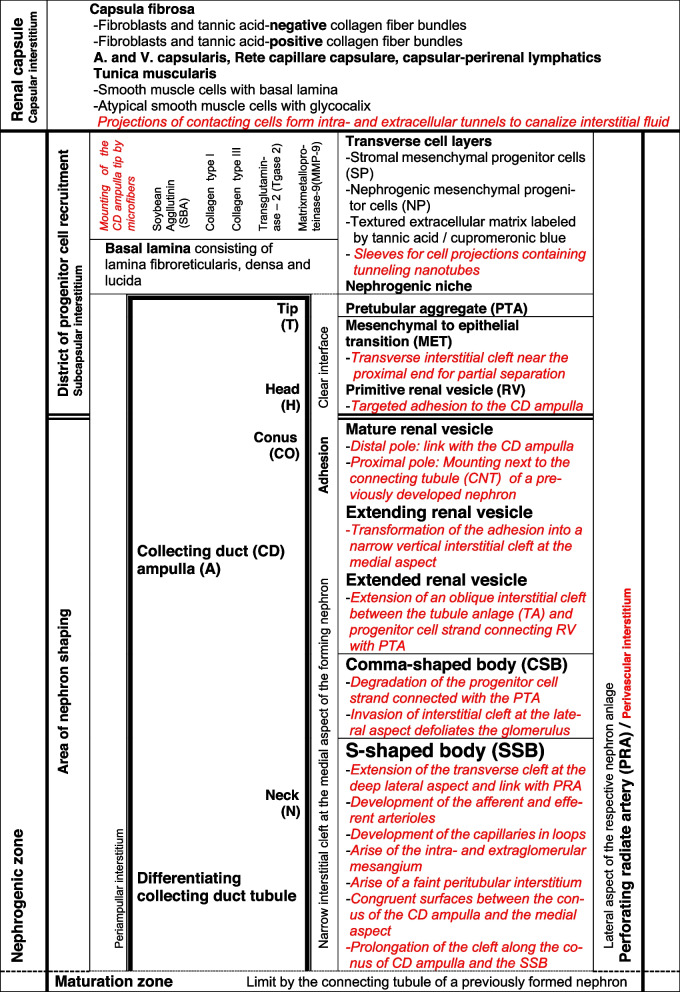
List of prominent installation sites (red color/cursive) at the forming nephron in the fetal human kidney during advanced pregnancy. The nephrogenic compartment is marked by a thick black line, and the collecting duct (CD) ampulla by a thick/thin line. The transverse double line at the section border between the head and conus of the CD ampulla separates the district of progenitor cell recruitment from the area of nephron shaping

The development of a nephron in the nephrogenic compartment correlates with a radial expansion of the width in the nephrogenic zone. In order to recognize the current status, one must recognize not only the shapes and individual positions of the nephron stages, but also the borders of a nephrogenic compartment (Table 1, Fig. 2). While the renal capsule covers the top, the border at the medial side is limited by a vertically lining CD ampulla. The border at the lateral side is a perforating radiate artery, which ascends towards the renal capsule. The base of a nephrogenic compartment is at the connecting tubule of a previously developed nephron. Restricted to this site, the future proximal pole of the currently forming nephron is mounted. The space between the presently available stage of nephron anlage and its structural neighbors reflects the respective extension of the interstitium.

The mounting of the proximal pole at the primitive renal vesicle next to the connecting tubule of a previously formed nephron defines the base position of the currently forming nephron. In contrast, at the distal pole and at both sides, the relation to the structural neighbors is altering during the further developmental steps. To explain these circumstances, a transverse line is drawn at the section border between the head and conus of the related CD ampulla so that a nephrogenic compartment is subdivided into an upper and a lower part. In each of them, quite different compounds are contained (Fig. 2). The upper district of progenitor cell recruitment is represented during the development from the primitive renal vesicle to the S-shaped body by a prone rectangle, which maintains its size. Only here, the tip of the CD ampulla with the integrated epithelial progenitor cells is present. This faces the inner layer of the competent nephrogenic mesenchymal progenitor cells. At the top, the renal capsule covers all. More laterally from the tip, the pretubular aggregate stays positioned at the head of the CD ampulla (Figs. 2 and 3). Only at the proximal end of the pretubular aggregate, the mesenchymal to epithelial transition takes place. Very impressive, solely at this site, the primitive renal vesicle will appear. Meanwhile, its establishing proximal pole is mounted next to the underlying connecting tubule of a previously developed nephron. Thus, the space of the primitive renal vesicle reflects the claim of the henceforth opening area of nephron shaping. During the further eccentric but radial development of the nephron anlage, the area of nephron shaping is recognized as an expanding quadrate. At this site, the mature, extending, and extended renal vesicle (Figs. 5, 6 and 7), the comma- (Fig. 8), and the S-shaped body (Fig. 9) stages will find the necessary space for their spatial expansion.

### Renal capsule covers the installation

In each of the nephrogenic compartments, the externally situated district of progenitor cell recruitment is covered by the renal capsule (Table 1, Figs. 2 and 3). By the first view, it looks rather unspectacular. The externally situated capsula fibrosa of the renal capsule consists of several strata, which protects the expanding outer cortex of the fetal human kidney during advanced pregnancy against inappropriate influences. Visible are numerous fibroblasts between the layers of collagen fiber bundles. Further on, at the outer surface and inside the capsula fibrosa arterial, venous, and lymphatic vessels occur. Most relevant for the nephron installation are the here ending perforating radiate arteries. Each of them lines vertically through the nephrogenic zone towards the renal capsule. Before, it establishes the link with the arising glomerular tuft at the deep lateral aspect of the S-shaped body. The internally situated tunica muscularis of the renal capsule represents the outer border of the nephrogenic zone. When it is regarded by the optical microscope, length- and crossway-sectioned smooth muscle cells are here recognized. Especially noticeable is that these face the underlying pool of interstitial progenitor cells without showing a recognizable compartmentalization.

### Meeting at the nephrogenic niche

Regarding orientated sections by the optical microscope, the distance between the inner side of the renal capsule and the tip of a CD ampulla is unexpectedly small. In average, it shows a vertical width of only 30 μm. One layer of interstitial (stromal) mesenchymal progenitor cells faces the inner side of the renal capsule, while one to two layers of the nephrogenic mesenchymal progenitor cells can be recognized at the CD ampulla tip (Fig. 3). Further one can recognize that these do not touch, instead they are separated by a clear interface.

### Pretubular aggregate

While a series of morphogenic proteins is reciprocally exchanged in the nephrogenic niche, the nephrogenic mesenchymal progenitor cells translocate during induction from the tip to the lateral edge of the related CD ampulla. Opposite the head of the CD ampulla, they become part of the pretubular aggregate (Figs. 2 and 3). This shows a tear drop-like form and exhibits a specific spatial but resting installation. Its thin distal end is orientated towards the renal capsule so that it remains connected with the nephrogenic progenitor cells, which are located beyond the tip of the CD ampulla. In contrast, the broad proximal end of the pretubular aggregate is positioned next to the connecting tubule of a previously developed nephron. Its medial part has a slightly concave form, which faces the head of the related CD ampulla. Surprisingly, between both of the structures, a clear interface is visible. The lateral part of the pretubular aggregate exhibits a convex surface, which borders the subcapsular interstitium at the inner side of the renal capsule.

Most pivotal for the installation of the presumptive nephron is the proximal end of the pretubular aggregate. The lateral part of its proximal end is positioned near the perivascular interstitium of a vertically lining perforating radiate artery. The mid of its proximal end is mounted next to the connecting tubule of a previously developed nephron. Especially remarkable, the medial part of its proximal end adheres at the section border between the head and upper conus of the related CD ampulla. As a result, the clear interface between the medial part of the pretubular aggregate and the head of the CD ampulla is terminated at this site. This is a most important step in the installation process, since it reflects the connection between the future connecting tubule of the currently forming nephron and the CD ampulla.

### Mesenchymal to epithelial transition and primitive renal vesicle

A next important step of the installation at the forming nephron is the mesenchymal to epithelial transition (MET; Fig. 3). It is restricted to the proximal end of the pretubular aggregate. This again is exclusively positioned at the transverse section border between the head and the upper conus of the involved CD ampulla.

The mesenchymal to epithelial transition results in the formation of the primitive renal vesicle (Fig. 4). It is first recognized as an open clamp at the proximal end of the pretubular aggregate. Later, the clamp forms a circle so that the small lumen of the primitive renal vesicle is enclosed by a single-layered polarized epithelium. For the moment, only the lateral part of the distal pole at the primitive renal vesicle remains connected with the overlying pretubular aggregate. However, the medial part of the distal pole shows a partial separation. In this situation, the lateral aspect of the primitive renal vesicle faces the perivascular interstitium of a here ascending perforating radiate artery. At the medial aspect of the primitive renal vesicle, the adhesion at the section border between the head and conus of the CD ampulla is replaced by a lasting attachment. Very special, the proximal pole of the primitive renal vesicle remains mounted by the installation next to the connecting tubule of a previously developed nephron.

### Mature, extending, and extended renal vesicles

Rather hidden but most decisive is the installation during development of the mature (Fig. 5), extending (Fig. 6), and extended (Fig. 7) renal vesicle stages. These solely expand in the area of nephron shaping.

### Proximal-distal positioning

While the coordinates of the primitive renal vesicle reflect the starting claim of the shaping nephron (Fig. 4), its proximal-distal orientation is determined by the expansion of the mature renal vesicle (Fig. 5). This is achieved by the solid mounting of the proximal pole (prospective Bowman’s capsule) in the pendentive, which is formed between the connecting tubule of an earlier formed nephron and the lower part of the conus at the CD ampulla. In so far, the proximal pole represents a fixed point. In contrast, the distal pole of the renal vesicle marks the mobile point, which will translocate in vertical (radial) direction. Due to its only partial separation from the pretubular aggregate, the lateral part of the distal pole is still connected via a two-layered progenitor cell strand. In contrast, the medial part of the distal pole remains attached at the section border between the head and the upper part of the conus at the CD ampulla.

### Partial separation from the pretubular aggregate

The actual separation of the mature (Fig. 5), extending (Fig. 6), and extended (Fig. 7) renal vesicle stages from the overlying pretubular aggregate occurs in a stepwise de-installation by an unknown molecular mechanism. At the primitive renal vesicle, very first signs are recognized as a narrow transverse cleft at the end of the clear interface and the section border between the head and upper conus of the CD ampulla (Fig. 4). While the mature renal vesicle develops, the separation transversely continues up to the mid of the pretubular aggregate (Fig. 5). In the extending (Fig. 6) and extended (Fig. 7) renal vesicle stages, only the lateral part of the distal pole remains connected with the pretubular aggregate via the two-layered progenitor cell strand.

### Link with the CD ampulla

Between the medial aspect of the mature renal vesicle and the upper conus of the CD ampulla, a striking interaction is registered. Following the development of the primitive (Fig. 4), mature (Fig. 5), extending (Fig. 6), and extended (Fig. 7) renal vesicle stages, one can see that the initial adhesion at the section border between the head and conus of the CD ampulla is replaced by an attachment. Later, at this site, the invasion of the future connecting tubule leads to a lasting physiological link. For this interactive installation, it is indispensable that the facing basal laminae of both the renal vesicle and the CD ampulla dissolve so that the different epithelia can fuse. It is unknown by which molecular mechanism the local interstitium performs this striking linking.

### Closeness replaced by distance

While the renal vesicle stages develop, the medial part of the distal pole represents the future connecting tubule, which is linked with the CD ampulla at the section border between its head and upper conus (Figs. 5, 6 and 7). In contrast, the lateral part of the distal pole is still connected with the overlying pretubular aggregate via the two-layered progenitor cell strand. Underneath the distal pole, as well as the tubule anlage, the progenitor cell strand vertically extends. Caused by this, the interstitial cleft between the tubule anlage and the progenitor cell strand changes direction first from transverse to oblique and then to vertical. In the interior of the renal vesicle, the inner epithelial fold protrudes towards the lumen of the vesicle. This happens near the underlying proximal pole, which is mounted opposite the connecting tubule of a previously developed nephron. Between the further elongating conus of the CD ampulla and the medial aspect of the in parallel extending renal vesicle, a surprising closeness is visible. However, during the ongoing development, the plaque of adhesion is replaced by a narrow interstitial cleft. In contrast, the entire lateral aspect of the extended renal vesicle is averted from the CD ampulla. Instead, it is exposed to the perivascular interstitium of a neighboring perforating radiate artery.

### Common radial expansion

While the renal vesicle stages develop, the CD ampulla radially elongates (Figs. 5, 6 and 7). Thereby, the future connecting tubule at the distal pole of the renal vesicle becomes successively integrated at the section border between its head and conus. The proximal pole of the respective renal vesicle stays mounted next to the connecting tubule of an earlier developed nephron. In this microanatomical constellation, the medial aspect of the developing renal vesicle is very close at the conus of the CD ampulla. When the conus of the CD ampulla elongates, due to the mounting at the both poles, this evokes tension at the body of the renal vesicle. In turn, it enables a stepwise vertical elongation at its middle portion. Thinkable is also that the spatial expansion of the renal vesicle is the driving force, which supports the vertical elongation at the conus of the CD ampulla. In each case, the expansion of the renal vesicle in concert with the elongation at the conus of the CD ampulla causes that beside the tip and head of the CD ampulla, also the overlying pool of progenitor cells, the pretubular aggregate, and the covering renal capsule are radially lifted to the same extend.

### Comma-shaped body

A special challenge for the process of installation is the comma-shaped body (Fig. 8). During this important developmental phase, not only basic interior changes such as the vertical elongation of the tubule anlage and the extension of the medial, inner, and lateral folds but also structural alterations of the shape at its outer side are most relevant.

At the medial part of the distal pole, the comma-shaped body is yet linked via its forming connecting tubule with the CD ampulla at the section border between its head and conus. Very decisively for the future development, at the lateral part of the distal pole, the earlier mentioned progenitor cell strand is degrading so that the recruitment of progenitor cells from the pretubular aggregate to the comma-shaped body is henceforth lost. Consequently, the interstitium located between the connecting tubule and the disappearing progenitor cell strand yet fuses with the perivascular interstitium of the neighboring perforating radiate artery. This causes that the contour at the distal pole is represented solely by the connecting tubule and the elongating distal tubule.

Particularly striking, at the lateral aspect of the comma-shaped body, precisely between its lateral fold and the elongating tubule anlage, a vertically lining interstitial cleft opens. During the preceding development, it changes direction from vertical to transverse, so that the opening finally becomes positioned at the deep lateral aspect of the comma-shaped body. The tip of the interstitial cleft ends at the turn up of the inner fold. This again indicates that the glomerulus is defoliating at the proximal pole. While a smaller part of the future Bowman’s capsule (lateral leg of the lateral fold) faces the perivascular interstitium at the perforating radiate artery, the major part represents the proximal pole of the comma-shaped body. It stays mounted next to the connecting tubule of a previously developed nephron.

The earlier described narrow and parallel lining course between the medial aspect of the late comma-shaped body and the conus of the CD ampulla is now replaced by a narrow interstitial cleft. At the confluence between the future connecting tubule and the conus of the CD ampulla, this turns direction to follow the elongating tubule portions towards the interior of the comma-shaped body.

### S-shaped body

The S-shaped body develops as the last transient stage of nephron anlage. Meanwhile, the physiological connection between the currently developing connecting tubule and the CD ampulla at the section border between its head and conus is completed (Fig. 9). Although it looks unspectacular, the pendentive between the currently completing connecting tubule and the head of the CD ampulla is most relevant for a potential further nephron installation. At the top, the pendentive is bordered by the proximal end of the overlying pretubular aggregate and a small part of the associated subcapsular interstitium. The lateral border is formed by the perivascular interstitium of a perforating radiate artery. When the process of installation supports it, within this narrow pendentive, a new primitive renal vesicle can be released to develop in the henceforth opening area of nephron shaping.

A further striking feature is present between the medial aspect of the S-shaped body and the conus of the CD ampulla. Although a narrow and congruent course exists between both structures, an interjacent interstitial cleft lines up to the connecting tubule. At the meeting with the connecting tubule, the cleft turns direction to extend along the meandering distal, intermediate, and proximal tubule portions. Here, it meets the newly formed peritubular interstitium of the currently developing S-shaped body.

In contrast to the medial aspect, at the lateral aspect of the S-shaped body, the microanatomical configuration is completely different. The outer contour of the connecting and distal tubule portions faces the perivascular interstitium of the related perforating radiate artery. However, at the deep lateral aspect of the S-shaped body, a transverse cleft is seen, which is situated between the overlying proximal tubule portion and the subjacent visceral podocyte cell layer of the arising glomerulus. The position of the cleft opening clearly depicts the invasion of interstitial cells, which perform the link between the perivascular interstitium of the perforating radiate artery and the arising glomerular tuft. Consequently, inside this cleft as well as the afferent and efferent arterioles, the capillary loops as the intra- and extraglomerular mesangium will develop.

Underneath the transverse cleft at the lateral aspect of the S-shaped body, the glomerulus is establishing. One can recognize the layer of podocytes, the Bowman’s space, and the externally situated Bowman’s capsule. It represents the proximal pole of the S-shaped body, which is mounted next to the connecting tubule of an earlier developed nephron.

The progress of installation in the late S-shaped body is recognized by a further vertical extension and segmentation along its proximal-distal axis. At its proximal pole, the typical morphological features of the future glomerulus become visible. The Bowman’s capsule flattens, the Bowman’s space is clearing, and the podocytes achieve their typical cobblestone-like shape. Due to the parallel elongation of the CD ampulla, the medial part of the Bowman’s capsule becomes positioned near its neck. The meandering tubule portions of the S-shaped body are located opposite the conus of the CD ampulla. Henceforth, the maturation of the nephron starts. As a consequence, the S-shaped body is adopted by the maturation zone.

### Installation requires nutrition and respiratory gas

The numerous developmental activities in the nephrogenic zone of the fetal human kidney during advanced pregnancy require beside a continuous provision with nutrition and respiratory gas also an elimination of the metabolic waste. The current problem is that not a single systematic investigation exists, which is dealing with this subject.

**Fig. 10:**
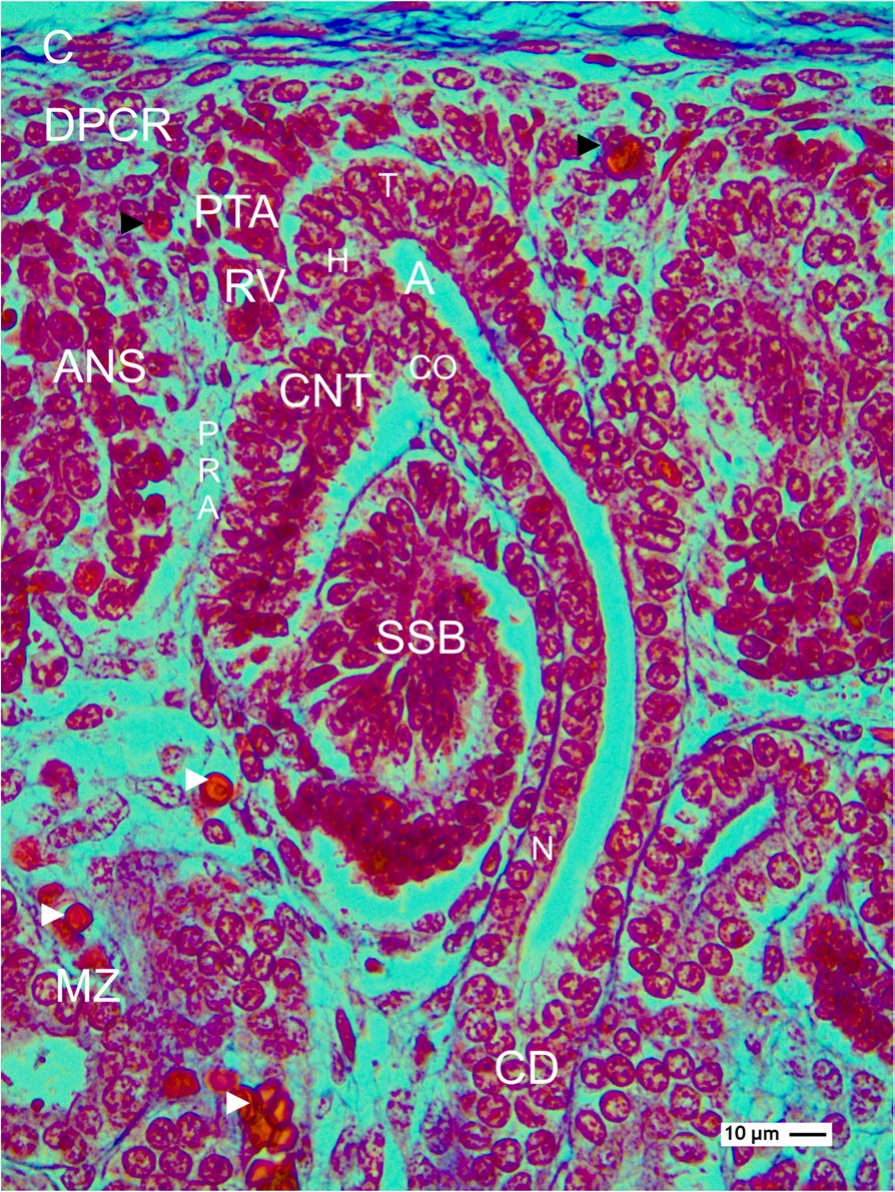
View onto the occurrence of erythrocytes in the fetal human kidney during advanced pregnancy by the optical microscope. The specimen stained by Azan depicts that microvessels perfused by erythrocytes are restricted to the district of progenitor cell recruitment (DPCR, black arrow heads) of the nephrogenic zone and the maturation zone (MZ, white arrow heads). In contrast, in the area of nephron shaping (ANS) including the vertically lining perforating radiate artery (PRA), the occurrence of erythrocytes is not observed. C renal capsule, CD differentiating collecting duct tubule, T tip, H head, C conus of the A collecting duct ampulla, PTA pretubular aggregate, RV primitive renal vesicle, CNT connecting tubule, SSB S-shaped body

Regarding the microscopic specimens, it is surprising that circulating blood cells—as an indicator for perfused vessels—are registered only in the vessels of the renal capsule, in the district of progenitor cell recruitment of the nephrogenic zone, and in the maturation zone (Fig. 10). In contrast, in the area of nephron shaping, some microvessels are observed; however, it seems that these are not yet perfused by erythrocytes. Although the perforating radiate arteries, the afferent and efferent arterioles, and the capillaries of the forming glomerulus at the late S-shaped body become visible, the lack of visible circulating blood cells indicates bradytrophic physiological conditions and hypoxia at this site of the nephrogenic zone. Consequently, the needed nutrition and respiratory gas for the developing nephron anlage stages including their installation must be delivered in incomplete paths via the interstitial fluid. It appears that 3 different options for the fluid input are probable.

First, the generated data depicts that a perforating radiate artery lines along the lateral aspect of a shaping nephron, which includes the development from the primitive renal vesicle up to the S-shaped body (Fig. 10). While these stages of nephron anlage are subject of a vertical and transverse extension, it is most probable that the neighboring perforating radiate artery elongates in parallel according to the developmental progress of the nephron. One could speculate that the perforating radiate artery temporarily and only in the elongating segment reveals leaky characteristics. In this situation, the contained serum including nutrition and respiratory gas may leave the lumen to penetrate the vessel wall and to reach the interstitial fluid in the perivascular space. Due to its topological proximity, especially the lateral aspect of the shaping nephron would profit from fresh nutrition and respiratory gas.

Second, the rete capsulare of the renal capsule provides beside the externally situated capsula fibrosa also the internally located tunica muscularis. Strikingly, at the meeting between the inner side of the tunica muscularis and the outer side of the nephrogenic zone, where the pool of progenitor cells is bordering, a prominent vascularization is lacking (Fig. 10). Instead, the interstitial fluid seems to be distributed between the here located smooth muscle cells and fibroblasts in lacunae-like fluid paths. It is thinkable that due to pressure in and on the vessels of the renal capsule, the distributing interstitial fluid including fresh nutrition and respiratory gas is reaching by this way as well as the underlying pool of progenitor cells, the nephrogenic niche, the pretubular aggregate, and the mesenchymal to epithelial transition as the distal pole of the renal vesicle.

Third, a series of microvessels containing blood cells is visible at the transition between the inner side of the nephrogenic zone and the underlying maturation zone (Fig. 10). Consequently, it is imaginable that here the interstitial fluid arises, which flows around the respective proximal pole and medial aspect of the overlying renal vesicle, comma-, and S-shaped body stages.

## Discussion

### Installation starts near the inner side of the renal capsule

The initial formation of new nephrons in the fetal human kidney during advanced pregnancy is restricted to the nephrogenic zone, which extends as a thin stripe of about 150 μm vertical width along the renal capsule [7, 30]. The actual development starts in the small space between the inner side of the renal capsule and the tip of a CD ampulla, which exhibits an average vertical width of only 30 μm (Fig. 3) [16]. At this site, an unexpected structuring exists [31].

Information from the fetal human kidney is not available. However, from the neonatal rabbit kidney, it is known that the collagen in the externally situated capsula fibrosa is specifically stacked and that only the internal collagen bundles are contrasted by tannic acid, which indicates a special micromilieu [32]. In the underlying tunica muscularis beside typical, also atypical smooth muscle cells are contained [33]. Only a part of these cells is covered by a basal lamina, while the others exhibit a faint glycocalyx. Most interestingly, at the transition between the inner side of the renal capsule and the external part of the nephrogenic zone, the here occurring cells send out numerous projections, so that the interjacent space represents a complex tunnel system. Since it faces the underlying pool of progenitor cells, it may serve the distribution of nutrition and respiratory gas via the interstitial fluid.

For both the fetal human and neonatal rabbit kidney, it was further shown that microfibers, which bind for example soybean agglutinin (SBA), originate at the inner side of the renal capsule, vertically cross the transverse interstitial and nephrogenic mesenchymal progenitor cell layers for the link with the tip of a CD ampulla [34]. Label for the extracellular matrix-stabilizing tissue transglutaminase 2 (Tgase2) and the matrix degrading metalloproteinase 9 (MMP9) in the neonatal rabbit kidney revealed that the mounting by the microfibers is not static but that it is subject of a conversion, which depends on the current branching of the CD ampulla [35].

### Installation takes place at structured sites

The generated data also depicted that the nephrogenic niche is restricted to the tip of the related CD ampulla, while the pretubular aggregate including the mesenchymal to epithelial transition remains positioned at its head (Fig. 3). Thereby, only the medial aspect of the pretubular aggregate faces a clear interface. Typically, this is terminated at the section border between the head and upper conus of the related CD ampulla. The here presented results clearly show that exactly at this site, the distal pole of the evolving primitive renal vesicle will adhere (Fig. 4).

Unfortunately, not for the head but solely for the tip of the CD ampulla, which represents the epithelial part of the nephrogenic niche, first information dealing with the clear interface is available (Fig. 3). In the optical microscope, one can recognize that the cell bodies of the innermost layer of the nephrogenic mesenchymal progenitor cells and the facing epithelial progenitor cells, which are contained in the tip of a CD ampulla, obviously do not touch (Figs. 3, 4, 5, 6, 7, 8 and 9). Astonishingly, for the neonatal rabbit kidney, it was shown by transmission electron microcopy that the distance is caused by the striking basal lamina covering the tip of a CD ampulla. Here, for example, P_CD_ Amp 1 as a site-specific molecule was detected [36]. Improved contrasting of specimens by tannic acid or cupromeronic blue further exhibited that the extracellular matrix at the interface is specifically textured [37]. For the nephrogenic mesenchymal progenitor cells, it was shown that these attach and detach from the CD ampulla tip across time [38]. However, it was also observed that the resting nephrogenic mesenchymal progenitor cells send out projections, which are covered by a special sleeve of extracellular matrix [39]. This construction enables that the cell projections of the nephrogenic mesenchymal progenitor cells cross the interface, penetrate the basal lamina, and contact via tunneling nanotubes the basal plasma membrane of the epithelial progenitor cells, which are contained in the tip of a CD ampulla. Less considered, but decades ago, comparable cell contacts were demonstrated in the nephrogenic niche of the embryonic mouse kidney [40]. In related transfilter culture experiments, it was further shown that intact cell projections, cell to cell contacts, and at least 24 h of a continuous contact are needed for a successful induction of the nephrogenic progenitor cells [41].

Thus, the data indicates that the meeting at the nephrogenic niche between the competent nephrogenic mesenchymal progenitor cells and the epithelial progenitor cells, which are contained in the tip of the related CD ampulla, is neither accidental nor brief but that it is subject a sophisticated and extended installation. This enables not only a targeted docking but it also serves the nephrogenic mesenchymal progenitor cells to linger for the controlled exchange of morphogenic proteins. It is obvious that this basic process requires a certain period of time, since morphogenic proteins not only with good (GDNF, FGF8) and minor (BMP4, BMP7) but also with poor (Wnt4, Wnt5a, Wnt 9b, Shh) solubility have to be exchanged [42]. It is most probable that also the cells in the neighboring pretubular aggregate profit from this construction.

### Installation proceeds along a guide rail

**Fig. 11:**
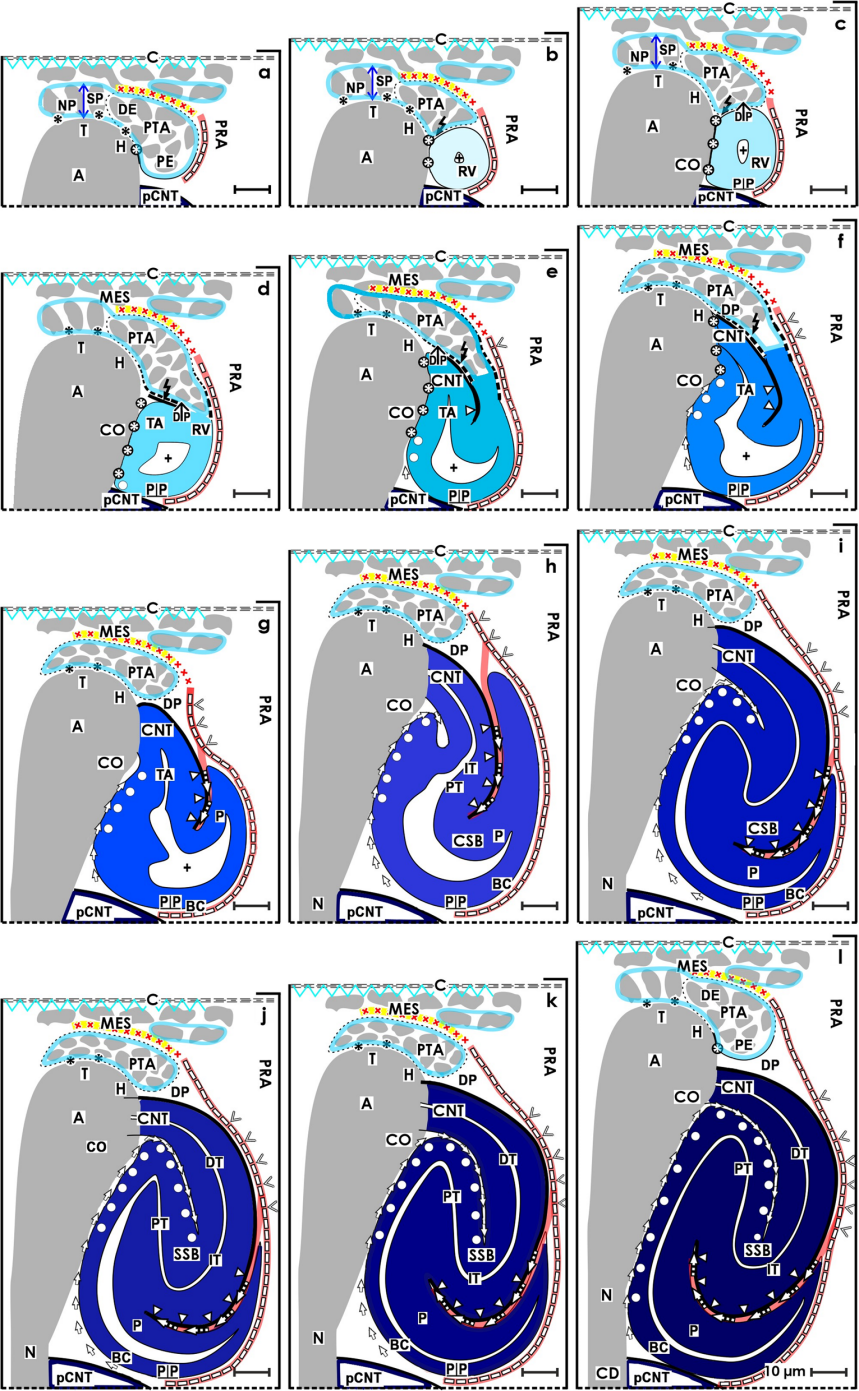
**a**–**l** Sketches almost true to scale allocate the installation at the transient stages of nephron anlage in the human fetal kidney during advanced pregnancy to the different compartments of the local interstitium. This is represented by the interstitum within the renal capsule (light blue/zig-zag), the interstitium between the inner side of the renal capsule and the tip of a CD ampulla (dark blue/vertical double arrow), the subcapsular interstitium (yellow line with crosses), the perivascular interstitium at the perforating radiate artery (PRA, white boxes with red line), the peritubular interstitium at the connecting tubule (black line), the interstitial cleft at the deep lateral aspect between the tubule anlage and the defoliating glomerulus during formation of the comma- and S-shaped bodies (white boxes with red line and arrow head), the oblique interstitial cleft between the tubule anlage and the progenitor cell strand, which connects the distal pole up to the extended renal vesicle stage with the proximal end of the pretubular aggregate (dashed black line). Visible is the precise site of the (**a**) nephrogenic niche, the pretubular aggregate (PTA), the (**b**) mesenchymal to epithelial transition, and the (**c**) primitive renal vesicle (RV). Shown are further the renal capsule (C), stromal progenitor cells (SP), nephrogenic progenitor cells (NP), distal end (DE), and proximal end (PE) of the pretubular aggregate, clear interface (black asterisks), connecting tubule (pCNT) of a previously developed nephron, collecting duct (CD) ampulla (A), tip (T), head (H), conus (CO), neck (N), adhesion of PTA (white asterisks) at the section border between the head and conus of CD ampulla, partial separation (flash) of the RV from PTA and lumen (**+**). Further are seen: the (**d**) mature, (**e**) extending, and (**f**) extended renal vesicles. Visible is the distal pole (DP), proximal pole (PP), tubule anlage (TA) with the future connecting tubule of the currently developing nephron. The white arrow heads point to progenitor cell strand connecting RV with PTA. The white circles indicate the congruent relation between the medial aspect of the forming nephron and the conus of the CD ampulla. In the (**g**) early, (**h**) mid, and (**i**) late comma-shaped bodies (CSB) are shown the distal tubule (DT), intermediate tubule (IT), proximal tubule (PT), future podocytes (P), and presumptive Bowman’s capsule (BC). In the the (**j**) early, (**k**) mid, and (**l**) late S-shaped bodies (SSB) the white triangles indicate the defoliation () of the glomerulus at the proximal pole. It initiates the physiological connection between the perforating radiate artery and the arising glomerular tuft. As a result, the darkening of color at the arising nephron stages coincides with the expanding progress of installation at quite different sites

The present investigation depicts that the transient stages of nephron anlage such as the nephrogenic niche, pretubular aggregate, mesenchymal to epithelia transition (Fig. 3), renal vesicles (Figs. 4, 5, 6, and 7), the comma- (Fig. 8), and S-shaped (Fig. 9) bodies are not distributed at random but within predictable coordinates. During the developmental progress, the forming nephron increases in size, evolves proximal and distal poles, and takes asymmetrical shapes at its medial and lateral aspects. The here presented photographic illustrations and sketches true to scale demonstrate that the morphological chances in the forming nephron appear in a certain extend autonomically, but to a certain degree, these arise in parallel with the establishing structural neighbors (Figs. 3, 4, 5, 6, 7, 8 and 9, 11), in particular with the different compartments of the local interstitium (Table 2). Most prominent is the guide rail for the forming nephron. It starts in the district of progenitor cell recruitment between the inner side of the renal capsule and the tip of a CD ampulla with the nephrogenic niche (Fig. 3). It proceeds along the head of the CD ampulla by a definitive positioning of the pretubular aggregate and at its proximal end with the punctual arise of the mesenchymal to epithelial transition. While the primitive renal vesicle develops, it adheres at the section border between the head and upper conus of the CD ampulla (Fig. 4). Finally, the mature (Fig. 5), extending (Fig. 6) and extended (Fig. 7) renal vesicles, the comma- (Fig. 8), and S-shaped (Fig. 9) bodies demonstrate a close and surprisingly congruent course between their respective medial aspects and the elongating mid and lower conus of the CD ampulla.

**Table 2:**
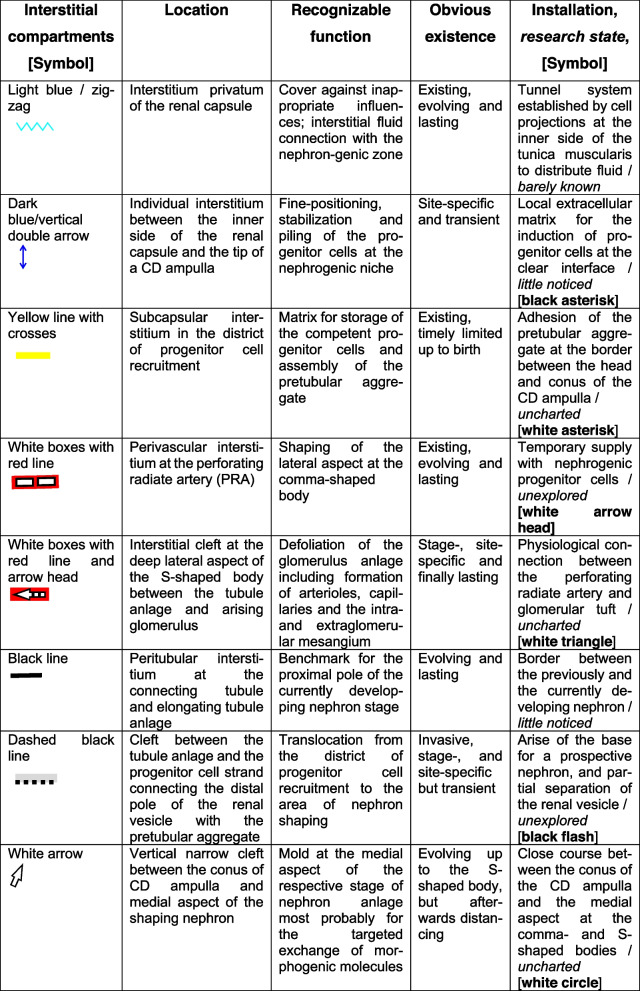
Allocation of prominent steps of installation to the different interstitial compartments at the forming nephron in the fetal human kidney during advanced pregnancy. It is demonstrated that quite different installation activities take place in the different interstitial compartments

Furthermore, it was demonstrated that the proximal pole of the currently shaping nephron remains positioned next to the connecting tubule of an earlier formed nephron (Figs. 4, 5, 6, 7, 8 and 9). Consequently, this mounting indicates the fix point. In contrast, the distal pole is connected with the CD ampulla at the section border between its head and upper conus. Thus, in the case the CD ampulla elongates, the distal pole represents the vertically shifting mobile point (Fig. 11 d–l). This again exerts tensile stress between the poles; at the same, it enables the spatial expansion and promotes the internal folding while the comma and S-shaped bodies develop. In particular, it serves the opening of the cleft at the deep lateral aspect at the S-shaped body so that the microvascularization at the defoliating glomerulus can establish.

### Installation manages different key points

At the head of the CD ampulla, the pretubular aggregate stays positioned for the entire period of nephron formation (Fig. 3). At its proximal end, first the mesenchymal to epithelial transition, then the arise of the primitive renal vesicle, and finally its only partial separation take place (Fig. 4). These decisive steps of installation raise the up to date unanswered questions, whether the production of a renal vesicle at the proximal end of the pretubular aggregate is principally a single event or whether the repetitive formation is possible [43]. Regarding the microscopic specimens, it is not answered, in how far only in the present section plane or whether also in the anterior or posterior planes of the pretubular aggregate further renal vesicles are formed so that possibly several nephrons are formed in a circumference around the head of a CD ampulla.

After the separation of the primitive renal vesicle, a further important step of the installation takes place at the section border between the head and upper conus of the related CD ampulla. Up to date, it is unclear why exactly at this site and by which molecular mechanisms first the adhesion of the distal pole at the primitive renal vesicle takes place, which leads then to an attachment and finally to the lasting link via the future connecting tubule [44–47]. Unknown is also why at the same time and by which kind of molecular linking the proximal pole of the renal vesicle is mounted next to the connecting tubule of an underlying but previously developed nephron.

Obvious is that the installation has essential consequences for the further development. When the distance between the fixed proximal and the mobile distal poles increases during the ongoing spatial expansion, the respective medial aspect of the extending renal vesicle stages up to the S-shaped body is bordered by the elongating conus of the CD ampulla. In contrast, the lateral aspect of these stages represents an open flank, which faces the interstitium of an ascending perforating radiate artery. Surprisingly, restricted to the deep lateral aspect, solely here the link between the tuft of the defoliating glomerulus and the ascending perforating radiate artery is observed. Unknown is whether the asymmetrical medial and lateral aspects of the shaping nephron are covered by a different composition of extracellular matrix [48], whether the site-specific installation is controlled by different matrix metalloproteinases [49] and whether the before described noxae impairing nephrogenesis can eventually cause at this site an imbalance of these regulating enzymes.

### Installation as a very likely target of noxae impairing nephrogenesis

The shaping nephron including the renal vesicle stages, comma-, and S-shaped bodies as the related CD ampulla consists each of a single-layered polarized epithelium, which is covered by a basal lamina (Figs. 4, 5, 6, 7, 8, 9 and 10). Consequently, the related lamina fibroreticularis is linked with the extracellular matrix of the local interstitium [20]. Considering both the microanatomical conditions and the here passing flow of interstitial fluid, one recognizes that not the shaping nephron or the CD ampulla but the interjacent interstitium and the here running process of installation are exposed in the first place, to the highest concentrations and for most of the time to noxae impairing nephrogenesis (Table 2).

However, there might be gradual differences in the distribution of noxae, when these are provided by the interstitial fluid (Fig. 10, Table 2). Considering both the laws of molecular diffusion and the local microanatomical conditions, the extended surface at the lateral aspect, the proximal, and the distal pole of the shaping nephron appear to be most exposed to the noxae. In contrast, the medial aspect of the shaping nephron, which is located next to the conus of the CD ampulla, appears to be less exposed due to the high resistance of fluid flow in a narrow interstitial cleft.

When the focus is directed to the numerous enzymes and complex molecular interactions, which build up and degrade the extracellular matrix in the interstitium, it is most probable that the noxae impairing nephrogenesis first target at this balance before they affect the developing nephron as it was shown in the fetuses of diabetic rats [49, 50]. Since data for preterm and low birth weight babies is lacking, the task for the future is to elaborate, whether for example the expression of matrix metalloproteinases (MMPs) and related tissue inhibitors (TIMPs) are very first influenced by noxae. In addition, information must be generated in how far the interstitial cells are sensitive against a chronical exposure of the mentioned noxae impairing nephrogenesis, and whether thereby the piloting functions of the interstitium are negatively interfered. Even important is to investigate, whether individual molecules of the noxae are metabolized in the interstitium so that they become cytotoxic only for the interstitial cells, for the progenitor cells, or only for distinct epithelial cell types of the shaping nephron.

## Conclusions

The present investigation suggests that the search for initial traces left by noxae impairing nephrogenesis should not focus alone on the forming nephron. Even important are the structural neighbors such as the collecting duct (CD) ampulla and the perforating radiate artery. However, of special relevance is the interjacent interstitium with its piloting and integrative functions, and the here running process of installation. This concerns crucial developmental stations such as the targeted meeting of progenitor cells in the nephrogenic niche, the only partial separation of the primitive renal vesicle from the pretubular aggregate, the linking of the shaping nephron via the connecting tubule with the CD ampulla, or the vascular connection between the tuft of the defoliating glomerulus and the neighboring perforating radiate artery. It must be accepted that due to its location, the local interstitium at the forming nephron and the here running successive series of installation is exposed from the onset to the highest concentrations of noxae impairing nephrogenesis.

## Data Availability

The data sets generated and/or analyzed during the current study are not public available due to ongoing research but are available from the corresponding author on reasonable request.
